# Comparison of first-generation EGFR-TKIs combined with low-dose bevacizumab versus osimertinib in untreated advanced EGFR-mutated NSCLC

**DOI:** 10.1080/07853890.2025.2493766

**Published:** 2025-04-25

**Authors:** Yingqi Xu, Yidan Zhang, Hongping Jin, Hua Zhong, Jianlin Xu, Yuqing Lou, Runbo Zhong

**Affiliations:** Department of Respiratory and Critical Care Medicine, Shanghai Chest Hospital, Shanghai Jiao Tong University School of Medicine, Shanghai, China

**Keywords:** Bevazicumab, EGFR-TKI, low-dose, NSCLC, osimertinib

## Abstract

**Background:**

This study aimed to compare the efficacy of first-generation epidermal growth factor receptor (EGFR)-tyrosine kinase inhibitors (TKIs) combined with low-dose bevacizumab(7.5 mg/kg) versus osimertinib as first-line treatment in patients with advanced EGFR-mutated non-small cell lung cancer (NSCLC).

**Materials and methods:**

A total of 161 patients with advanced NSCLC harboring EGFR mutations, who received first-line treatment at Shanghai Chest Hospital between July 2017 and July 2023, were enrolled in this study. Among them, 78 patients were treated with a combination of first-generation EGFR-TKIs and bevacizumab (7.5 mg/kg), constituting the bevacizumab plus TKI (A + T) group. The remaining 83 patients received osimertinib monotherapy (80 mg daily), forming the osimertinib group.

**Results:**

The objective response rate (ORR) was 65.4% (51/78) in the A + T group and 68.7% (57/83) in the osimertinib group (*p* = 0.657). Despite the potentially poorer baseline conditions of patients in the osimertinib group, the median progression-free survival (PFS) was 16.59 months (95% CI: 14.39–18.80) in the A + T group compared to 16.82 months (95% CI: 13.76–19.89) in the osimertinib group (*p* = 0.792). Preliminary overall survival (OS) analysis indicated a median OS of 51.75 months (95% CI: 41.63–61.86) in the A + T group versus 35.55 months (95% CI: 22.32–48.77) in the osimertinib group (*p* = 0.010), however, the OS data are not yet mature.

**Conclusion:**

Although osimertinib remains the preferred first-line treatment for advanced NSCLC with EGFR mutations, combining first-generation EGFR-TKIs with low-dose bevacizumab may be a viable alternative for certain patients.

## 1. Introduction

Lung adenocarcinoma (LUAD) is one of the most common types of non-small cell lung cancer (NSCLC). Approximately 11% of Caucasian patients and 50% of Asian patients with LUAD harbor epidermal growth factor receptor (EGFR) mutations, with exon 21 L858R and exon 19 deletions (19del) being the most prevalent variants [1]. All three generations of EGFR-tyrosine kinase inhibitors (TKIs) are approved for first-line treatment in patients with advanced EGFR-mutated NSCLC [2–6]. Osimertinib, a third-generation EGFR-TKI, has demonstrated significantly longer progression-free survival (PFS) compared to first-generation TKIs (18.9 months vs. 10.2 months) in the FLAURA trial [6], establishing it as the preferred first-line option.

However, patients with the L858R mutation appear to derive less OS benefit from osimertinib [7]. Furthermore, first-line osimertinib-treated patients from Asia demonstrate less OS benefit compared to non-Asian patients [8]. Therefore, we may need to explore more treatment strategies for these patients.

Upregulation of EGFR signaling in EGFR-mutated NSCLC increases VEGF expression, contributing to resistance to EGFR-TKIs *via* hypoxia-independent mechanisms [9,10]. Preclinical studies have demonstrated that combining anti-VEGF therapy with EGFR-TKIs exerts synergistic antitumor effects, potentially overcoming resistance [11]. In contrast to the suboptimal outcomes observed with third-generation EGFR-TKIs combined with bevacizumab [12], patients receiving first-generation EGFR-TKIs combined with bevacizumab demonstrated superior PFS compared to those on first-generation EGFR-TKI monotherapy. The median PFS for first-generation EGFR-TKIs combined with bevacizumab ranged from 15.4 to 17.9 months [13–16], with a median OS of 33.3 to 50.7 months in patients with advanced EGFR-mutated NSCLC [13,14,16,17]. These outcomes are comparable to those achieved with first-line osimertinib. Subgroup analyses indicated that Asian patients with the L858R mutation derived favorable benefits from this combination therapy [13,15].

The AVAIL trial [18] demonstrated that low-dose bevacizumab(7.5 mg/kg) combined with chemotherapy provided similar survival benefits and reduced toxicity compared to the standard 15 mg/kg dose in advanced NSCLC patients. This finding suggests that low-dose bevacizumab combined with first-generation EGFR-TKIs could offer a more practical and tolerable treatment option in real-world clinical settings.

This study aimed to compare treatment outcomes between first-generation EGFR-TKIs combined with low-dose bevacizumab and osimertinib monotherapy in previously untreated patients with advanced EGFR-mutated NSCLC. The objective was to determine whether the combination of first-generation EGFR-TKIs and low-dose bevacizumab could serve as a viable alternative to osimertinib for first-line treatment in this patient population.

## 2. Methods

### 2.1. Patients

We retrospectively reviewed the medical records of 11,696 patients with EGFR-mutated NSCLC treated at Shanghai Chest Hospital between July 2017 and July 2023.

Eligibility criteria included: (I) patients with pathologically confirmed advanced NSCLC; (II) patients harboring EGFR-sensitizing mutations, specifically exon 19 deletions or exon 21 L858R mutations; and (III) patients treated with either first-generation EGFR-TKIs combined with low-dose bevacizumab or osimertinib monotherapy as first-line therapy.

Exclusion criteria were: (I) patients with other malignancies, (II) patients treated with first- or second-generation EGFR-TKI monotherapy or combination therapies excluding bevacizumab, and (III) patients without complete follow-up data.

The eligible patients were grouped into first-generation EGFR-TKIs combined with low-dose bevacizumab group (TKI + Avastin, A + T group) and osimertinib monotherapy group.

### 2.2. Clinical assessments and follow-up

Staging assessments were conducted in all patients at initial diagnosis according to the 8th edition of the tumor node metastasis (TNM) classification system. Imaging techniques were used to evaluate treatment efficacy throughout the treatment period until the termination of treatment. Chest computed tomography (CT) scan was performed at two to three monthly intervals to assess treatment efficacy. Additional imaging modalities such as abdominal ultrasound, cranial magnetic resonance imaging (MRI), and bone emission computed tomography (ECT)were added if required. These assessments continued until either disease progression, therapy termination or the last follow-up visit, whichever occurred first.

The best objective responses (BORs) were evaluated according to response evaluation criteria in solid tumors (RECIST v1.1), including complete response (CR), partial response (PR), stable disease (SD), or progressive disease (PD). Objective response rate (ORR) was defined as the proportion of patients achieving CR or PR radiologically. The disease control rate (DCR) was defined as the proportion of patients who achieved CR, PR and SD based on imaging assessments. The PFS was calculated from the beginning of treatment to disease progression, treatment alteration due to intolerance of treatment, death, or the last follow-up visit, whichever came first. The OS was calculated from the beginning of treatment to death or the last follow-up visit, whichever came first. Adverse events (AEs) were categorized according to the Medical Dictionary for Regulatory Activities (MedDRA v20.0) and graded according to the common terminology criteria for adverse events (CTCAE v5.0).

The key assessment indicators included ORR, DCR, PFS, OS and AEs. The last follow-up visit was on September 20, 2024.

### 2.3. Statistical analysis

Statistical analyses were performed using SPSS 20.0 statistical software (IBM, Armonk, NY, USA). The age of patients was presented as the median and range, while other patient characteristics were presented as categorical variables, indicating the number and percentage of patients. Chi-square test was used to compare BORs. Kaplan–Meier method and the log-rank test were used to estimate and compare survival outcomes (PFS and OS). Multivariate Cox regression was used to identify significant factors related to PFS and OS. Data were presented as median values with their respective 95% confidence intervals (CIs). *p* ≤ 0.05 was considered statistically significant.

## 3. Results

### 3.1. Baseline characteristics

**Figure 1.**
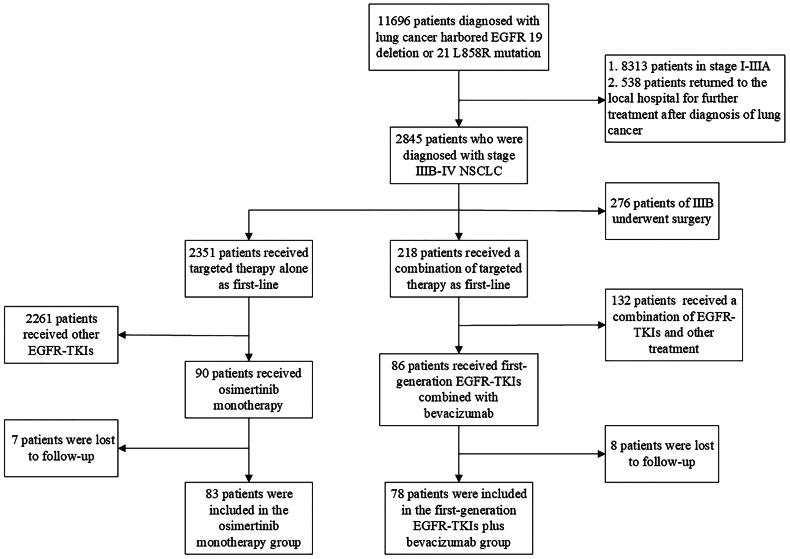
CONSORT diagram.

At the initial diagnosis, 2,845 of 11,696 patients were diagnosed with stage IIIB–IV EGFR-mutated NSCLC. Of these patients, 86 received first-generation EGFR-TKIs combined with low-dose bevacizumab as first-line treatment, while 90 received osimertinib. In the A + T group and osimertinib monotherapy groups, eight and seven patients were lost to follow-up, respectively. Thus, 78 and 83 patients were included in the A + T and osimertinib monotherapy groups, respectively (Figure 1).

Demographic and baseline characteristics of patients in each group are shown in Table 1. The patient population consisted predominantly of women (54.7%) and non-smokers (79.5%), and the most common histological type was adenocarcinoma (99.4%). Among the observed EGFR mutations, 51.6% were identified as EGFR exon 19 deletion, while 48.4% were identified as EGFR exon 21 mutation. In the A + T group, 7.7% were classified as stage IIIB, 2.6% were stage IIIC, 37.2% were stage IVA, and 52.6% were stage IVB. In the osimertinib monotherapy group, 3.6% were classified as stage IIIB, 34.9% were stage IVA, and 61.4% were stage IVB. Table 2 shows the targeted therapeutic drugs administered to patients in the A + T group. The patients in the A + T group received bevacizumab 7.5 mg/kg.

**Table 1.** Demographic and baseline characteristics of patients who received A + T or osimertinib monotherapy.

Characteristics	A + T group (*n* = 78) (%)	Osimertinib group (*n* = 83) (%)
***Gender***		
Male	31 (39.7)	42 (50.6)
Female	47 (60.3)	41 (49.4)
***Median age, years(range)***	64.0 (27.0–79.0)	63.0 (39.0–83.0)
***Smoking status***		
Non-smokers	58 (74.4)	70 (84.3)
Smokers	20 (25.6)	13 (15.7)
***ECOG PS score***		
0	2 (2.6)	4 (4.8)
1	52 (66.7)	52 (62.7)
2	24 (30.8)	27 (32.5)
***Pathological type***		
Adenocarcinoma	78 (100.0)	82 (98.8)
Non-adenocarcinoma	0 (0.0)	1 (1.2)
***Method of preoperative diagnosis***		
EBUS-TBNA	14 (17.9)	20 (24.1)
TBB/TBLB	17 (21.8)	17 (20.5)
CT-guided percutaneous lung puncture	11 (14.1)	15 (18.1)
Supraclavicular lymph node biopsy	21 (26.9)	22 (26.5)
Pleural effusion embedding	15 (19.2)	9 (10.8)
***Clinical stage***		
IIIb	6 (7.7)	3 (3.6)
IIIc	2 (2.6)	0 (0.0)
Iva	29 (37.2)	29 (34.9)
IVb	41 (52.6)	51 (61.4)
***T stage(before treatment)***		
T1a	0 (0.0)	0 (0.0)
T1b	1 (1.3)	1 (1.2)
T1c	15 (19.2)	12 (14.5)
T2a	16 (20.5)	19 (22.9)
T2b	10 (12.8)	16 (19.3)
T3	15 (19.2)	14 (16.9)
T4	21 (26.9)	21 (25.3)
***N stage***		
N0	10 (12.8)	6 (7.2)
N1	6 (7.7)	7 (8.4)
N2	29 (37.2)	13 (15.7)
N3	33 (42.3)	57 (68.7)
***Brain metastases***		
Yes	21 (26.9)	35 (42.2)
No	57 (73.1)	48 (57.8)
***Bone metastases***		
Yes	40 (51.3)	56 (67.5)
No	38 (48.7)	27 (32.5)
***Liver metastases***		
Yes	3 (3.8)	13 (15.7)
No	75 (96.2)	70 (84.3)
***EGFR-activating mutations***		
Exon 19 deletion	35 (44.9)	48 (57.8)
Exon 21 mutation	43 (55.1)	35 (42.2)

**Table 2.** Targeted therapeutic drugs administered to patients in the A + T group.

EGFR-TKIs	*N* (*n* = 78) (%)
Gefitinib	16 (20.5)
Erlotinib	31 (39.7)
Icotinib	31 (39.7)

The median follow-up time in our study was 39.62 months. In the A + T group, the median treatment duration was 16.2 months (95% CI: 15.9–21.4 months), while in the osimertinib monotherapy group, it was 16.8 months (95% CI: 16.1–21.2 months).

At the data cutoff, 130 patients had disease progression in the two groups: 63 (80.8%) in the A + T group and 67 (80.7%) in the osimertinib group. Of the 78 patients who received A + T, the following achieved the BORs: 51 patients achieved PR, 26 patients achieved SD, and one patient achieved PD. Two of these patients switched their regimens after three and four months due to abnormal liver function; they achieved PR and SD before being excluded from the study. Of the 83 patients in the osimertinib group, the following achieved the BORs: 57 patients achieved PR, 24 patients achieved SD, and two patients achieved PD. Two of these patients switched their regimens after one month due to mouth ulceration and abnormal liver function, respectively, and they achieved SD before being excluded from the study (Table 3). The ORR was 65.4% (51/78) in the A + T group and 68.7% (57/83) in the osimertinib group (*p* = 0.657). The DCR was 98.7% (77/78) in the A + T group and 97.6% (81/83) in the osimertinib group (*p* = 0. 597).

**Table 3.** The BORs of patients who received A + T or osimertinib monotherapy.

	A + T group(*n* = 78) (%)	Osimertinib group (*n* = 83) (%)
Complete response	0 (0.0)	0 (0.0)
Partial response	51 (65.4)	57 (68.7)
Stable disease	26 (33.3)	24 (28.9)
Progression disease	1 (1.3)	2 (2.4)

**Figure 2.**
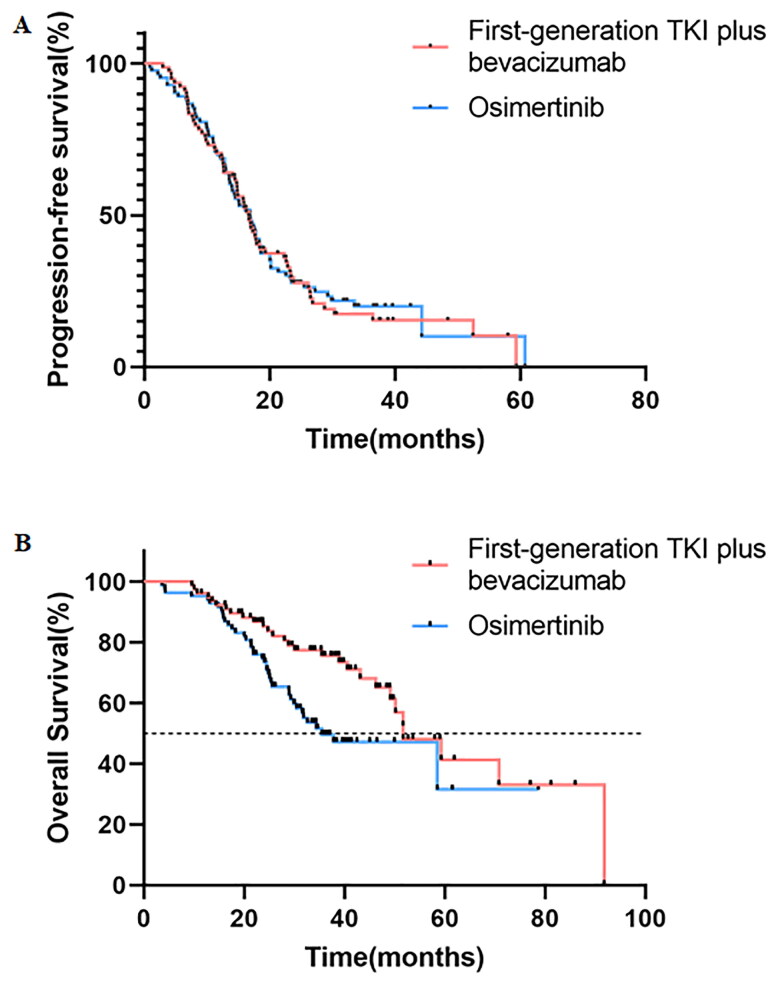
Kaplan–Meier analysis of (A) PFS of all patients, (B) OS of all patients. PFS, progression-free survival; OS, overall survival.

The median PFS in the A + T group was 16.59 months (95% CI: 14.39–18.80), while it was 16.82 months (95% CI: 13.76–19.89) in the osimertinib group (*p* = 0.813) (Figure 2A), and no significant difference was observed. The PFS rates at 12 months in the A + T and osimertinib groups were 69.2% and 68.7%, respectively.

At the final data cutoff, 68 deaths were reported, comprising 28 (35.9%) in the A + T group and 40 (48.2%) in the osimertinib group. The median OS in the A + T group was 51.75 months (95% CI: 41.63–61.86), compared to 35.55 months (95% CI: 22.32–48.77) in the osimertinib group (*p* = 0.010) (Figure 2B), although these results are not yet mature. The OS rates at 12 months were 94.9% for the A + T group and 94.0% for the osimertinib group. After two years, the OS rates were 85.2% for the A + T group and 73.4% for the osimertinib group, and at three years, the rates were 75.6% and 49.7%, respectively.

**Figure 3.**
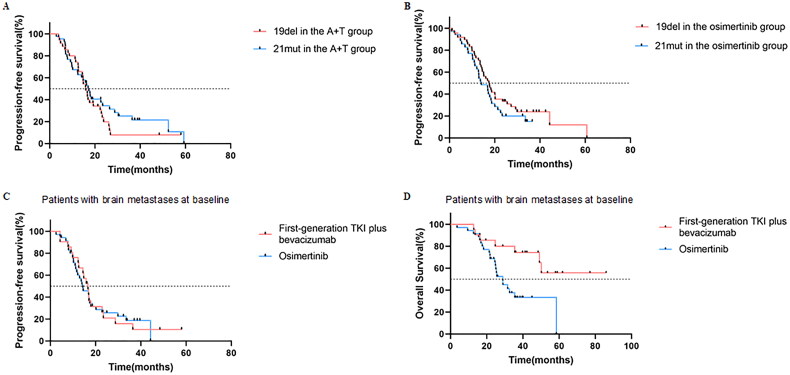
Kaplan–Meier analysis of (A) PFS of patients with different mutations in A + T group, (B) PFS of patients with different mutations in osimertinib group, (C) PFS of patients with brain metastases at baseline, (D) OS of patients with brain metastases at baseline. PFS, progression-free survival; OS, overall survival.

No statistically significant differences were observed in PFS between patients harboring EGFR 19del (15.87 months, 95% CI: 13.66–18.08) and EGFR 21 L858R (17.25 months, 95% CI: 14.74–19.77) in the A + T group (*p* = 0.287). The corresponding difference was not observed in the osimertinib group (17.41 vs. 14.03 months) (95% CI: 13.85–20.98; 95% CI: 9.38–18.68) (*p* = 0.252) (Figure 3A and 3B).

Median PFS in the A + T-treated patients with brain metastases was 16.59 months (95% CI: 13.52–19.66), while in the osimertinib-treated patients, it was 14.32 months (95% CI: 9.79–18.85) in (*p* = 0.948) (Figure 3C). The median OS of A + T-treated patients with brain metastases was not reached, while it was 28.91 months (95% CI: 23.08–34.74) in osimertinib-treated patients. However, a significant difference was observed (*p* = 0.008) (Figure 3D).

**Figure 4.**
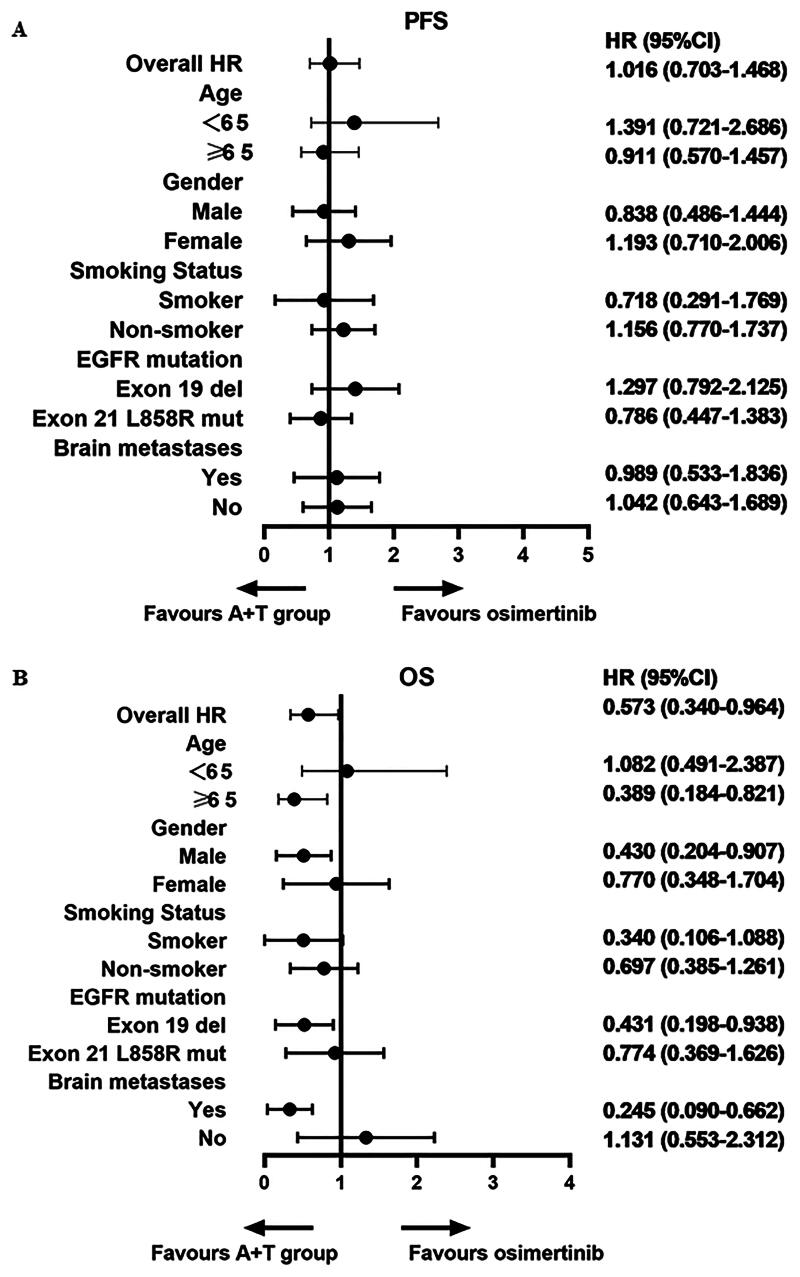
Multivariate cox regression related to (A) PFS and (B) OS. PFS, progression-free survival; OS, overall survival.

Subgroup analyses of PFS revealed no significant differences based on age (<65 vs. ≥65 years), sex (male vs. female), smoking status (non-smoker vs. smoker), or EGFR mutation type (exon 19 deletion vs. exon 21 L858R mutation) between the two groups. However, subgroup analysis of OS indicated that patients with EGFR 19del mutations and those with baseline brain metastases may benefit more from A + T compared to osimertinib. Figure 4 shows the forest plot for subgroup analysis.

In the A + T group, 74.6% (47/63) of patients received subsequent anticancer treatment after first-line progression. Among those who received second-line treatment, 76.6% (36/47) continued with targeted therapy, of which 88.9% (32/36) were treated with osimertinib as their second-line option. Of these 32 patients, 15 developed T790M mutations identified through tissue re-biopsy or blood tests, while 17 transitioned to osimertinib without undergoing genetic testing. The remaining 23.4% (11/47) received chemotherapy or chemotherapy combined with other treatments after progression. Among the 63 patients who experienced disease progression, 37 underwent re-biopsy during second-line or subsequent treatment, including 27 who had tissue re-biopsies (with 13 confirming T790M positive) and 10 who had peripheral blood genetic testing (with 5 confirming T790M positive).

In the osimertinib group, 67 patients experienced disease progression. Among them, 65.7% (44/67) received second-line treatment, with 19.4% (13/67) continuing TKI monotherapy. Of the remaining patients, 17 underwent chemotherapy, 3 received immunotherapy, and 11 were treated with a combination of chemotherapy and either targeted therapy or immunotherapy.

### 3.2. Safety

The most common AEs in the A + T group were rash (50.0%), diarrhea (35.5%), hypertension (32.9%), proteinuria (39.2%), anemia (34.2%), mouth ulceration (22.4%) and nausea (17.1%), shown in Table 4. The most common AEs in the osimertinib monotherapy group were rash (44.6%), diarrhea (32.5%), anorexia (32.5%), anemia (24.1%), mouth ulceration (32.5%), and paronychia (22.9%). Grade 3 toxicities occurred in 24 (30.8%) patients in the A + T group and 17 (20.5%) patients in the osimertinib monotherapy group. No grade 4 or 5 adverse events were reported.

**Table 4.** Adverse events during targeted therapy.

	Adverse events	CTCAE grade 1, *n* (%)	CTCAE grade 2, *n* (%)	CTCAE grade 3, *n* (%)	CTCAE grade 4, *n* (%)
A + T group (*n* = 78)	Rash	31 (39.7)	5 (6.4)	2 (2.6)	/
	Diarrhea	25 (32.1)	/	2 (2.6)	/
	Paronychia	12 (15.4)	/	/	/
	Hypertension	18 (23.1)	3 (3.8)	4 (5.1)	/
	Proteinuria	26 (33.3)	2 (2.6)	3 (3.8)	/
	Nausea	13 (16.7)	/	/	/
	Anemia	21 (26.9)	2 (2.6)	3 (3.8)	/
	Mouth ulceration	17 (21.8)	/	/	/
	Decreased WBC count	11 (14.1)	5 (6.4)	2 (2.6)	/
	Decreased platelet count	9 (11.5)	8 (10.3)	3 (3.8)	/
	Abnormal liver function	8 (10.3)	3 (3.8)	5 (6.4)	/
Osimertinib group (*n* = 83)	Rash	28 (33.7)	7 (8.4)	2 (2.4)	/
	Diarrhea	23 (27.7)	3 (3.6)	1 (1.2)	/
	Paronychia	19 (22.9)	/	/	/
	Anorexia	24 (28.9)	3 (3.6)	/	/
	Nausea	17 (20.5)	1 (1.2)	/	/
	Anemia	15 (18.1)	3 (3.6)	2 (2.4)	/
	Mouth ulceration	26 (31.3)	/	1 (1.2)	/
	Decreased WBC count	13 (15.7)	4 (4.8)	2 (2.4)	/
	Decreased platelet count	11 (13.3)	3 (3.6)	4 (4.8)	/
	Abnormal liver function	11 (13.3)	4 (4.8)	5 (6.0)	/

## 4. Discussion

We compared the efficacy of first-generation EGFR-TKIs combined with low-dose bevacizumab versus osimertinib monotherapy in untreated patients with advanced EGFR-mutated NSCLC. Although the baseline conditions of patients in the osimertinib group may have been poorer, we found no significant difference in PFS between the two groups (16.59 months vs. 16.82 months, *p* = 0.813). Early OS data suggest a trend favoring the A + T group, with a median OS of 51.75 months versus 35.55 months in the osimertinib group (*p* = 0.010), however, the OS data are not yet mature. Subgroup analyses suggested potential OS benefits for patients with EGFR 19del mutations or those with baseline brain metastases in the A + T group.

The predominant mechanism of resistance to first-generation EGFR-TKIs is the emergence of the T790M mutation. However, no biomarkers currently exist to accurately predict which patients will develop T790M following initial TKI resistance. Given that osimertinib was designed to target T790M-mediated resistance, it may be preferable as a first-line option for patients at high risk of developing this mutation following initial treatment with first-generation TKIs. However, in patients where T790M does not drive resistance to first-generation EGFR-TKIs, the benefit of using osimertinib as the initial therapy remains unclear. Previous studies of angiogenesis inhibitors combined with first-generation EGFR-TKIs have shown that combination therapy prolongs PFS compared to EGFR-TKI monotherapy, with a comparable incidence of acquired T790M mutations upon progression [15,19]. These findings suggest that adding an angiogenesis inhibitor to first-generation EGFR-TKIs does not prevent the emergence of the T790M resistance mechanism. Consequently, subsequent treatment with a T790M-targeting drug like osimertinib remains a viable next-line option. This rationale underpins the basis for our study.

We investigated whether OS could be enhanced by administering osimertinib to patients who developed a T790M mutation after initial treatment with A + T, as opposed to those who received first-line osimertinib. In this study, we identified 18 patients (13 through tissue re-biopsy, 5 through blood tests) in the combination therapy group with the T790M mutation after developing resistance to first-line A + T, comprising 28.6% (18/63) of the cohort—a notably lower proportion compared to previous studies [13,19]. The lower detection rate of T790M mutations in our cohort was largely due to 41.3% (26/63) of patients not undergoing genetic testing at the time of resistance. Many patients treated with first-line A + T were unable to undergo re-biopsy due to various reasons upon disease progression. Consequently, some individuals with acquired T790M mutations remained undetected and consequently missed the opportunity for osimertinib treatment. From this consideration, first-line osimertinib may be a more suitable approach.

Previous studies evaluated bevacizumab at 15 mg/kg in combination with first-generation EGFR-TKIs, while we used a lower dose of 7.5 mg/kg. According to the AVAIL trial [18], bevacizumab at 15 mg/kg combined with cisplatin–gemcitabine and bevacizumab at 7.5 mg/kg combined with the same regimen showed similar survival benefits in patients with advanced NSCLC, but with reduced toxicity at the lower dose. In this study, the combination of 7.5 mg/kg bevacizumab with first-generation EGFR-TKIs resulted in a median PFS of 16.59 months and an ORR of 65.4%, which were comparable to the median PFS of 16.82 months and ORR of 68.7% observed in the osimertinib monotherapy group. These findings suggest that a lower dose of bevacizumab combined with first-generation EGFR-TKIs may offer excellent therapeutic efficacy for patients with EGFR-mutant advanced NSCLC.

Patients with EGFR mutations derive greater benefits from osimertinib than from first-generation EGFR-TKIs monotherapy, regardless of smoking status, as previously reported [6]. However, the effectiveness of first-generation EGFR-TKI combined with bevacizumab in EGFR-mutated patients, based on smoking status, appears to vary among different ethnic groups. For Caucasians, smokers seem to benefit more from the combination therapy [16]. In contrast, in Asian populations, non-smokers tend to experience greater benefits [15], possibly due to population-specific factors. Caucasians who smoke may exhibit a higher prevalence of TP53 mutations, which are associated with increased VEGF expression in various solid tumors, including NSCLC [20]. As a result, VEGF or VEGF receptor inhibitors can improve outcomes in NSCLC patients with TP53 mutations [20,21]. The exon 21 L858R mutation, predominant in Asian non-smoking patients [22], often co-occurs with concurrent mutations [23], making these patients more likely to benefit from bevacizumab combined with EGFR-TKI therapy than from first-generation EGFR-TKI monotherapy. In this study, most Asian patients were non-smokers (74.4% in the A + T group and 84.3% in the osimertinib group), and the proportions of exon 19 deletion and exon 21 L858R mutation were similar. Despite smoking status, survival improvements were comparable for both treatments as first-line options in our study.

For patients with advanced NSCLC with central nervous system metastases, osimertinib had significantly longer PFS in the FLAURA study [6] than the first-generation TKI (HR: 0.47). Therefore, osimertinib was the preferred treatment option for patients with EGFR mutations with brain metastases. The limited therapeutic options beyond osimertinib suggested that alternative strategies are essential. In preclinical studies, bevacizumab prevents early angiogenesis, reduces the formation of brain metastases [24], and substantially reduces circulating S100A9-positive monocytic myeloid-derived suppressor cells (MDSCs). Circulating MDSCs could be recruited to the normal brain microenvironment, thus playing a critical role in brain metastatic niche formation [25]. The PFS of patients with EGFR-mutated NSCLC treated with first-generation TKI combined with bevacizumab varies in different studies. For example, the BEVERLY trial excluded patients with brain metastasis [16]. In contrast, studies such as NEJ026^15^ and ARTEMIS [13] reported PFS HRs of 0.78 and 0.48, respectively, for the A + T combination compared to first-generation TKI monotherapy. A retrospective study reported that first-generation TKI combined with bevacizumab could significantly improve the prognosis of patients with brain metastases than first-generation TKI monotherapy [26]. The results of our study showed a PFS HR of 0.989 in the direct comparison between A + T and osimertinib monotherapy, suggesting similar improvements for patients with brain metastases across both treatment strategies. Nonetheless, patients with brain metastases appeared to experience greater OS benefits with A + T (HR = 0.245) compared to osimertinib monotherapy. However, it’s important to note that the proportion of patients with brain metastases was higher in the osimertinib group (42.2%) than in the A + T group (26.9%). Additionally, these findings stem from an ad hoc analysis, and the OS data are not yet mature; therefore, these results should be interpreted with caution.

Patients with ex21 L858R benefit more from first-line osimertinib than first-generation TKI, based on PFS [6]. However, as the follow-up time extended, the PFS benefit did not translate into a significant OS benefit compared to first-generation TKI in the previous study [7]. According to preliminary OS data from three phase III studies, the therapeutic effect of A + T on exon 21 L858R mutations varied. In the BEVERLY trial, compared to first-generation TKIs, there was no significant improvement in PFS(HR: 0.75 [0.42–1.3]), and no improvement in OS (HR: 0.59 [0.29–1.18]) [16]. Conversely, the NEJ 026 trial showed benefits in PFS (HR: 0.57 [0.33–0.97]) [15], but not in OS (HR: 0.790 [95% CI: 0.460–1.358]) [17]. Similarly, in the ARTEMIS trial, improvements were seen in PFS (HR: 0.50 [0.32–0.77]), but OS did not show significant improvement (HR: 1.36 [95% CI: 0.53–3.47]) [13]. The aforementioned results indicate that the therapeutic benefits of A + T primarily manifest in PFS, particularly in Asian patients with exon 21 L858R mutations, with no significant improvement observed in OS. It’s worth noting that OS outcomes may have been influenced by subsequent therapies following disease progression, potentially affecting the interpretation of the impact of first-line treatment on OS. In direct comparison, this study demonstrated that A + T exhibited a comparable therapeutic effect to osimertinib in terms of PFS and OS for patients with exon 21 L858R mutations.

The toxicity in both groups was manageable and tolerable. The safety profile observed in this study was generally consistent with the known safety profiles of previous studies [6,12, 13,15]. Grade 3 AEs occurred among 24 (30.8%) of 78 patients in the A + T group and 17 (20.5%) of 83 patients in the osimertinib group. Other AEs, such as rash, diarrhea, paronychia, and nausea, were mild to moderate (grades 1–2), and the patients recovered regardless of medications.

At last, it is important to note that the cost of the A + T combination therapy does not offer an advantage over osimertinib, which may significantly limit its use as a first-line treatment.

There are several limitations to this study. Firstly, its retrospective nature inherently introduces selection bias. Secondly, the combination of low-dose bevacizumab (7.5 mg/kg) with EGFR-TKIs is not currently standard treatment due to a lack of large-scale prospective studies supporting its efficacy. Thirdly, patients who received the A + T combination therapy might generally have better overall physical condition, allowing them to tolerate the addition of anti-angiogenic agents from the beginning of treatment, which could potentially contribute to improved OS. Additionally, the osimertinib group in our study had a higher incidence of initial brain, liver, and bone metastases, suggesting a worse baseline condition. Therefore, caution should be exercised when interpreting the results. Additionally, the study’s follow-up duration was limited. It remains unclear whether A + T combination treatment would lead to a statistically improved OS with longer follow-up periods. Lastly, it is important to note that patient data collection relied solely on retrospective medical history descriptions, which may introduce subjective bias when assessing specific CTCAE grades.

## 5. Conclusion

While osimertinib remains the preferred first-line treatment for advanced NSCLC with EGFR mutations, combining first-generation EGFR-TKIs with low-dose bevacizumab may be a viable alternative for certain patients. Nevertheless, further research is needed to identify clinicopathological factors that may influence treatment selection.

## Data Availability

The data that support the findings of this study are available from the corresponding author upon reasonable request.
